# Supplementary material for: Bivalent COVID-19 mRNA booster vaccination (BA.1 or BA.4/BA.5) increases neutralization of matched Omicron variants
Source: NPJ Vaccines. 2023 Aug 4;8:110. doi: 10.1038/s41541-023-00708-9 (PMC10403593; doi:10.1038/s41541-023-00708-9)
Supplement: Supplementary file 1 — Supplements [file 41541_2023_708_MOESM1_ESM.pdf]

Supplementary Information:

Supplementary Table 1: Vaccination history of the monovalent vaccinated individuals

Study-ID (Person)	*Age (years)*	*Sex*	*1st vacc.*	*2nd vacc.*	*3rd vacc.*	*4th vacc.*	*Days 2nd vacc to 3rd vacc.*	*Days 3rd vacc to 4th vacc.*	*Days 3rd vacc. to blood sampling*	*Days 4th vacc. to blood sampling*
**P3**	54.3	f	P	P	P	N	285	N	26	N
**P4**	27.8	f	P	P	P	N	288	N	23	N
**P5**	47.9	f	P	P	P	N	287	N	20	N
**P9**	53.4	f	P	P	P	N	287	N	29	N
**P13**	32.6	f	P	P	P	N	290	N	26	N
**P14**	53.0	m	P	P	P	N	273	N	24	N
**P15**	45.9	f	P	P	P	N	287	N	24	N
**P36**	29.6	f	P	P	P	N	244	N	38	N
**P17**	46.8	f	P	P	M	N	224	N	38	N
**P19**	31.4	f	P	P	P	N	301	N	43	N
**P22**	32.4	f	P	P	M	N	224	N	28	N
**P23**	39.5	f	P	P	M	N	211	N	33	N
**P24**	37.2	f	P	P	M	N	224	N	28	N
**P25**	59.3	f	P	P	P	N	245	N	22	N
**P26**	26.3	f	P	P	P	N	268	N	21	N
**P37**	27.8	m	P	P	P	N	192	N	26	N
**P35**	27.0	m	P	P	P	N	278	N	23	N
**P1**	53	f	P	P	P	P	246	265	N	20
**P2**	42.2	m	P	P	P	P	287	245	N	16
**P42**	39.3	f	P	P	M	P	238	274	N	20
**P6**	50.1	f	P	P	M	P	235	287	28	20
**P8**	59.4	f	P	P	P	P	287	240	28	25
**P10**	37.6	f	P	P	P	P	286	265	21	23
**P11**	51.9	f	P	P	M	P	212	152	32	31
**P12**	57	f	P	P	P	M	163	175	40	29
**P16**	29.2	f	P	P	P	P	287	224	15	21
**P18**	52.7	f	P	P	M	P	238	300	22	30
**P21**	45.8	m	P	P	P	P	288	229	16	21
**P27**	64.8	f	P	P	P	P	287	156	30	22
**P28**	82.7	f	P	P	P	P	205	147	N	38
**P43**	80.1	m	M	M	M	M	NA	211	N	26
**P29**	56.3	m	P	P	P	P	245	262	N	24
**P38**	40.8	f	P	P	P	P	NA	231	N	21
**P39**	38.8	m	P	P	P	P	75	267	N	24
**P30**	54.6	f	P	P	P	M	251	251	30	30
**P44**	59.6	m	P	P	P	P	185	221	N	24
**P45**	59.2	f	P	P	P	P	128	228	N	22
**P46**	65.5	m	P	P	P	P	185	262	N	22
**P31**	56.1	f	P	P	P	M	252	263	32	24
**P32**	53.5	f	P	P	P	P	226	292	32	25
**P33**	62.4	m	P	P	P	P	283	241	21	21
**P34**	63.2	f	P	P	P	P	260	270	27	29
**P41**	58.3	f	P	P	P	P	180	274	N	18

Age: in years at time of blood sampling; m: male. f: female; vacc.: vaccination; P: Biontech/Pfizer “Comirnaty” BNT162b2; M: Moderna “Spikevax” mRNA-1273; NA: not available, N: No

Supplementary Table 2: Vaccination history of the bivalent vaccinated individuals

Study-ID (Person)	Age (years)	Sex	1st vacc.	2nd vacc.	3rd vacc.	4th vacc.	Days 2nd vacc to 3rd vacc.	Days 3rd vacc. to 4th vacc.	Days 4th vacc. to blood sampling
P89	39.4	m	AZ	AZ	P	P-BA.1	177	306	27
P90	52.8	f	P	P	P	P-BA.1	142	306	21
P91	54.3	f	P	P	P	P-BA.1	160	308	28
P92	38.1	m	P	P	P	P-BA.1	146	291	21
P93	62.2	m	P	P	P	P-BA.1	162	291	21
P94	52.7	m	P	P	P	P-BA.1	NA	NA	21
P95	51.4	f	AZ	AZ	P	P-BA.1	138	302	31
P96	44.73	w	J	P	P	P-BA.1	148	203	21
P97	40.10	m	P	P	P	P-BA.1	148	279	22
P98	58.92	m	P	P	P	P-BA.1	167	308	21
P99	54.54	m	P	P	P	P-BA.1	277	238	21
P100	46.43	w	P	P	P	P-BA.1	138	296	20
P101	39.1	f	P	P	P	P-BA.5	147	331	21
P102	46.4	m	P	P	P	P-BA.5	157	309	21
P103	38.6	m	J	P	P	P-BA.5	125	231	21
P104	34.4	m	P	P	P	P-BA.5	107	243	21
P105	56.0	f	J	P	P	P-BA.5	122	232	21
P106	58.4	f	P	P	P	P-BA.5	187	302	21
P107	52.6	f	AZ	AZ	P	P-BA.5	138	308	21
P108	62.5	m	P	P	P	P-BA.5	183	298	21
P109	52.1	f	P	P	P	P-BA.5	188	306	21
P110	41.9	m	J	P	P	P-BA.5	125	232	21
P111	38.8	f	P	P	P	P-BA.5	134	301	21
P112	32.8	f	P	P	P	P-BA.5	132	318	21
P113	55.9	f	P	P	P	P-BA.5	140	298	21
P114	45.0	f	P	P	P	P-BA.5	173	287	21
P115	57.3	f	P	P	P	P-BA.5	158	304	21
P116	50.1	f	P	P	P	P-BA.5	163	307	21
P117	56.2	f	P	P	P	P-BA.5	148	298	21
P118	50.7	f	AZ	AZ	P	P-BA.5	153	306	21
P119	55.7	m	J	P	P	P-BA.5	105	243	21
P120	57.0	m	P	P	P	P-BA.5	179	322	30
BT55	27.8	m	P	P	P	P-BA.5	278	329	28
P121	26.6	f	P	P	P	P-BA.5	NA	330	28

Age: in years at time of blood sampling; m: male, f: female; vacc.: vaccination; AZ: Astra-Zeneca "Vaxzevria" ChAdOx1; P: Monovalent Biontech/Pfizer "Comirnaty" BNT162b2; J: Johnson & Johnson Ad26.COV2.S; P-BA.1: Biontech/Pfizer Bivalent (WT/BA.1) BNT162b2 BA.1; P-BA.5: Biontech/Pfizer Bivalent (WT/BA.5) BNT162b2 BA.5; M: Moderna "Spikevax" mRNA-1273; NA: not available

Supplementary Table 3: Vaccination history of the subjects who experienced Omicron breakthrough infection

Study-ID (Person)	Age (years)	Sex	1st vacc.	2nd vacc.	3rd vacc.	4th vacc.	Days 2nd vacc to 3rd vacc.	Days 3rd vacc. to 4th vacc.	Infecting Omicron variant	Days last vacc. to infection	Days infection to blood sampling
**P09**	53.6	f	P	P	P	N	287	N	BA.1	101	20
**P17**	47.1	f	P	P	M	N	224	N	BA.1	143	19
**P22**	32.8	f	P	P	M	N	224	N	BA.1	139	18
**P23**	39.9	f	P	P	M	N	211	N	BA.1	142	18
**P52**	26.8	m	M	M	M	N	185	N	BA.1	28	20
**P53**	22.5	m	P	P	P	N	178	N	BA.1	35	20
**P54**	25.2	f	P	P	N	N	N	N	BA.1	202	17
**P55**	43.1	m	P	P	N	N	N	N	BA.1	123	13
**P56**	76.6	f	P	P	P	N	195	N	BA.1	110	30
**P57**	33.7	f	P	P	P	N	145	N	BA.1	105	36
**P59**	55.4	m	P	P	P	N	165	N	BA.1	99	24
**P04**	28.1	f	P	P	P	N	288	N	BA.2	110	22
**P28**	82.8	f	P	P	P	P	205	147	BA.2	80	16
**P37**	28.0	m	P	P	P	N	192	N	BA.2	59	28
**P48**	39.2	f	P	P	M	N	238	N	BA.2	245	56
**P49**	57.2	f	P	P	M	N	224	N	BA.2	190	27
**P51**	59.6	f	P	P	P	N	244	N	BA.2	179	35
**P58**	51.9	f	P	P	P	N	143	N	BA.2	114	27
**P03**	54.9	f	P	P	P	N	285	N	BA.4/5	218	24
**P14**	53.6	m	P	P	P	N	273	N	BA.4/5	216	28
**P19**	31.9	f	P	P	P	N	301	N	BA.4/5	199	24
**P24**	38.0	f	P	P	M	N	224	N	BA.4/5	321	30
**P25**	60.1	f	P	P	P	P	245	282	BA.4/5	13	22
**P27**	64.9	f	P	P	P	P	287	156	BA.4/5	40	36
**P29**	56.4	m	P	P	P	P	245	262	BA.4/5	50	23
**P44**	59.7	m	P	P	P	P	185	221	BA.4/5	38	24
**P47**	47.5	f	P	P	M	N	218	N	BA.4/5	285	52
**P50**	56.6	f	P	P	P	N	288	N	BA.4/5	212	22

Age: in years at time of blood sampling; m: male, f: female; vacc.: vaccination; AZ: Astra-Zeneca “Vaxzevria” ChAdOx1; P: Monovalent Biontech/Pfizer “Comirnaty” BNT162b2; P-BA.1: Biontech/Pfizer Bivalent (WT/BA.1) BNT162b2 BA.1; P-BA.5: Biontech/Pfizer Bivalent (WT/BA.5) BNT162b2 BA.5, M: Moderna “Spikevax” mRNA-1273; NA: not available, N: No
